# Effects of Heat-Killed *Lacticaseibacillus paracasei* MCC1849 on the Maintenance of Physical Condition in Healthy Adults: A Randomized, Double-Blind, Placebo-Controlled, Parallel-Group Study

**DOI:** 10.3390/nu15153450

**Published:** 2023-08-04

**Authors:** Soichiro Sato, Satoshi Arai, Noriyuki Iwabuchi, Miyuki Tanaka, Ryosuke Hase, Naoki Sakane

**Affiliations:** 1 Innovative Research Institute, R&D Division, Morinaga Milk Industry Co., Ltd., 5-1-83, Higashihara, Zama 252-8583, Japan; s-arai@morinagamilk.co.jp (S.A.); n-iwabuchi@morinagamilk.co.jp (N.I.); m_tanaka@morinagamilk.co.jp (M.T.); 2 Department of Public Health and Preventive Medicine, Yamaguchi University Graduate School of Medicine, 1-1-1, Minami-Kogushi, Ube 755-8505, Japan; hase@yamaguchi-u.ac.jp; 3 Division of Preventive Medicine, Clinical Research Institute, National Hospital Organization Kyoto Medical Center, 1-1, Fukakusa, Mukaihata-cho, Fushimi-ku, Kyoto 612-8555, Japan; nsakane@gf6.so-net.ne.jp

**Keywords:** *Lacticaseibacillus paracasei* MCC1849, postbiotics, subjective symptoms, immunity

## Abstract

We previously reported that the intake of heat-killed *Lacticaseibacillus paracasei* MCC1849 suppressed the onset of cold-like symptoms in healthy young women who were susceptible to colds. This study aimed to investigate the effects of MCC1849 on subjective symptoms of physical condition in healthy adults of a wide age range. In this randomized, double-blind, placebo-controlled, parallel-group study, 200 healthy adults were randomly divided into the MCC1849 group or placebo group. The participants received test powder with 50 billion MCC1849 cells or placebo powder without MCC1849 for 24 weeks. Subjective symptoms were assessed by diary scores. Analysis was performed on 183 participants (MCC1849 group; *n* = 91, placebo group; *n* = 92) in the per-protocol set. The number of days of stuffy nose and cold-like symptoms was significantly reduced in the MCC1849 group compared with the placebo group. In addition, the duration of stuffy nose, sore throat and cold-like symptoms was significantly lower in the MCC1849 group. No side effects were observed. Therefore, oral intake of MCC1849 suppressed subjective symptoms in healthy adults of a wide age range. These data suggest that MCC1849 may help maintain physical condition.

## 1. Introduction

The common cold is a mild upper respiratory illness caused by a variety of viruses and bacteria, including coronaviruses, rhinoviruses and adenoviruses [1]. The symptoms include local symptoms such as stuffy nose, cough and sore throat and systemic symptoms such as feverishness, chills and malaise. To prevent infectious diseases, vaccines are widely used to promote the production of antibodies against pathogens, and their effectiveness has been recognized [2]. However, the existence of multiple viruses that cause the common cold makes effective prevention by vaccines difficult [3]. Recently, COVID-19 has been spreading worldwide, but it is taking time to develop effective vaccines and drugs. The economic loss due to the common cold in the United States was estimated to be $40 billion per year [4], so it could be said that prevention of the common cold is a social issue.

Probiotics have been studied for their beneficial effects on immunity as functional foods. In particular, *Lactobacillus* and *Bifidobacterium* have been reported to suppress respiratory tract infection [5]. Furthermore, recent studies have indicated the functionality of heat-killed *Lactobacillus* and *Bifidobacterium* [6]. Heat-killed bacteria are one kind of postbiotic. Postbiotics are defined as preparations of inanimate microorganisms and/or their components that confer a health benefit on the host [7]. The bioactivities of postbiotics include immunostimulatory and anti-inflammatory effects [8]. Some heat-killed bacteria modulate the immune system. Therefore, the intake of heat-killed bacteria as postbiotics could be expected to maintain the physical condition.

Among other postbiotics, heat-killed *Lacticaseibacillus paracasei* MCC1849 (strain shield, hereinafter referred to as “MCC1849”) is known to modulate the immune system [9]. Oral administration of MCC1849 increased antigen-specific IgA levels in the small intestine, lung and serum in vivo [10]. In a clinical trial, MCC1849 intake enhanced the responsiveness of acquired immunity to influenza vaccination in people aged 85 years or older [11]. In healthy young women who were susceptible to cold, the onset of cold-like symptoms was suppressed by the ingestion of MCC1849 [12]. These clinical studies suggested an immunomodulatory effect of MCC1849, but these participants were elderly or young females. Age and sex seem to affect the responsiveness to vaccines, so these factors may affect the immune system [13]. Therefore, to clarify the effects on subjective symptoms of physical condition, we conducted this randomized, double-blind, placebo-controlled, parallel-group trial in both sexes of a wide age range.

## 2. Materials and Methods

### 2.1. Study Design and Statement of Ethics

We performed a randomized, double-blind, placebo-controlled, parallel-group trial in Yamaguchi Prefecture from December 2021 to May 2022. This study was registered in the UMIN-CTR (UMIN000045856) after we obtained approval from the Ethics Review Committee of the Japan Conference for Clinical Research, a nonprofit organization (approval code: 428, approval date: 10 September 2021). Before the study, its purpose, its procedures and the rights of the participants were fully explained to the participants, and written consent was obtained from all of them. The study was conducted in compliance with the Declaration of Helsinki (Fortaleza, revised in 2013) and the Ethical Guidelines for Life Sciences and Medical Research Involving Human Subjects (Ministry of Education, Culture, Sports, Science and Technology; Ministry of Health, Labor and Welfare; and Ministry of Economy, Trade and Industry Notification No. 1, 2021).

### 2.2. Participants

Participants were included if they were between the ages of 20 and 74 years. Participants with one or more of the following criteria were excluded from participation: (1) disease or serious medical history of the liver, kidney, heart, lung, gastrointestinal tract, blood, endocrine system or metabolism; (2) regular use of oral or nasal medications; (3) serious medication allergy, food allergy or history of any of these; (4) severe perennial or seasonal allergic symptoms; (5) plans to be vaccinated against COVID-19 from two weeks before the start of this study to the end of the study time when written consent was obtained; (6) pregnancy, lactation or expectation to be pregnant during this study; (7) participation in another study within 1 month prior to giving informed consent; and (8) any other reason making participation inappropriate as deemed by the principal investigator based on patient background and physical examination.

### 2.3. Intervention

The test powder was composed of 50 billion cells of MCC1849 and maltodextrin derived from tapioca starch as an excipient, and the placebo powder was maltodextrin without MCC1849. Test powder and placebo powder were produced by Morinaga Milk Industry (Tokyo, Japan). The taste, smell and appearance of the test foods were confirmed to be indistinguishable before and at the end of this study by an independent investigator. Participants received test powder or placebo powder with water once daily for 24 weeks.

We excluded participants who planned to be vaccinated against COVID-19, but did not prohibit vaccination during the intake period. If participants were vaccinated against COVID-19, the intake of test food was suspended for 5 days before and after vaccination. If a vaccinated participant did not suspend intake of the test food, data for the day of vaccination and the next 5 days were not used, as adverse reactions to the vaccine often occur during this time. The intake of supplements including *Lactobacillus*, *Bifidobacterium* or oligosaccharides was prohibited during this study. Participants were informed to refrain from consuming foods that affect the immune system.

### 2.4. Efficacy Assessment

The primary outcome of this study was subjective symptoms of physical condition. During the study period, participants were asked to record their severity scores in diaries for subjective symptoms such as feverishness, chills, malaise, joint pain, muscle pain, sneezing/runny nose, stuffy nose, sore throat and cough. Participants rated each symptom on a 0–4 severity scale: (0) none, (1) almost none, (2) a little, (3) moderate, and (4) severe. The total score was summed for all subjective symptoms for each day. The onset of each symptom was given a score of 3 or 4. The total number of days, duration, total number of times and incidence of each symptom during the intake period were calculated. As an exploratory assessment after unblinding, the onset of cold-like symptoms was defined as a total score of 6 or more. Overall cold-like symptoms were analyzed, as was each symptom.

The secondary outcome was febrile state. Participants recorded their body temperatures in their diaries. The number of days and participants with temperatures of 37.5 °C or higher during the intake period were calculated for each group.

### 2.5. Safety Assessment

All participants were monitored for adverse events and side effects throughout the 24-week intervention period and 1-week post-observation period. Safety monitoring included a questionnaire on general health and the occurrence of any health-related events. The severity of adverse events was evaluated according to the Common Terminology Criteria for Adverse Events version 4.0 JCOG/JSCO. The number of events and the incidence of occurrences were calculated for each group. The relationships between adverse events and intake of the test food were evaluated by the principal investigator.

### 2.6. Sample Size

For the incidence of the primary endpoint, the number of participants needed was calculated by G*power 3.1.9.7 (http://www.gpower.hhu.de/) accessed on 27 August 2021. The incidence of cold-like symptoms was assumed to be 45% in the placebo group and 34% in the MCC1849 group. In total, 200 participants were necessary to detect a difference between groups at a 5% significance level and an 80% statistical power considering a dropout rate of 20%.

### 2.7. Randomization

Participants were randomly divided into two groups in a 1:1 ratio using block randomization by an independent investigator. The stratification factor was the number of times they had contracted a cold in the past 3 years (0, 1–2, or more than 3). However, the allocation was performed by number of common colds in the past 3 years (0–1, 2, or more than 3), so there was a deviation from the protocol. The allocated sequence was sealed to both participants and investigators until the study ended and the database was locked.

### 2.8. Statistical Analysis

The primary population was the per-protocol set (PPS) population. The total number of days, the duration and the number of times of each symptom were compared between the placebo and MCC1849 groups using the Wilcoxon rank sum test. The incidence of each symptom was compared between groups using the χ^2^ test. The number and incidence of body temperature above 37.5 °C were compared between groups using the Wilcoxon rank sum test and the χ^2^ test, respectively. The incidence of adverse events and side effects was compared between the two groups using Fisher’s exact test. Two-sided *p* values less than 0.05 were considered to be statistically significant. All data were analyzed using IBM^®^ SPSS version 28.0 (IBM Corp, Armonk, NY, USA).

## 3. Results

### 3.1. Participants

**Figure 1:**
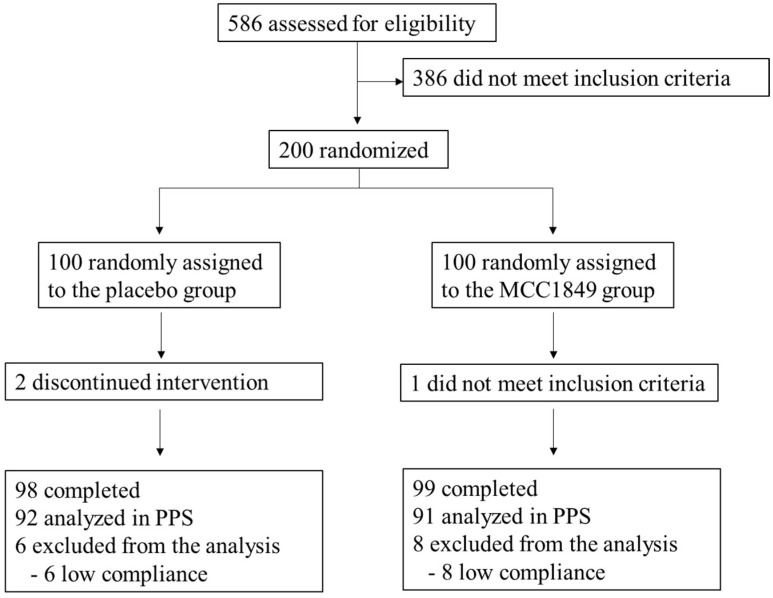
Study flow diagram.

Figure 1 shows the study flow diagram. During recruitment (October 2021–November 2021), 586 subjects were assessed for eligibility. A total of 200 participants were recruited to this study and allocated randomly into two groups. One participant in the MCC1849 group declined to participate due to not meeting the inclusion criteria before the start of this study. Therefore, the safety analysis set (SAF) included 199 participants who consumed the test food at least once. In the efficacy analysis, 14 participants were excluded due to low compliance: low intake of test powder or placebo powder (<70% in the test period: placebo, *n* = 2; MCC1849, *n* = 0), high intake of prohibited foods (placebo, *n* = 2; MCC1849, *n* = 3), or intake of drugs including antibiotics or remedies for the common cold (placebo, *n* = 4; MCC1849, *n* = 5). Thus, 183 participants were analyzed in the efficacy analysis (PPS). Some 122 participants (placebo, *n* = 63; MCC1849, *n* = 59) got vaccinated against COVID-19 during the study period.

### 3.2. Background

The background information is shown in Table 1. All participants were healthy and between the ages of 21 and 62. No differences in age, sex, BMI, COVID-19 vaccination during the study period or common cold in the past 3 years were detected between groups.

### 3.3. Effects on the Subjective Symptoms of Physical Condition

Table 2 compares the data of each symptom and the data of all cold-like symptoms between groups. Among the symptoms, the total number of days with stuffy nose and the average durations of stuffy nose and sore throat were significantly lower in the MCC1849 group than the placebo group (*p* < 0.05). The duration of feverishness tended to be lower in the MCC1849 group than in the placebo group (*p* < 0.1). Although there was no significant difference in the number of times or the incidence rate of each symptom, there was a trend toward fewer stuffy noses and sore throats in the MCC1849 group compared to the placebo group (*p* < 0.1). An exploratory evaluation of the cold-like symptoms also showed a significant reduction in their total number of days and in their duration in the MCC1849 group compared to the placebo group (*p* < 0.05).

### 3.4. Effect on the Febrile State

Table 3 compares the data of body temperature above 37.5 °C. There was no significant difference in the number of days or incidence between the placebo and MCC1849 groups.

### 3.5. Safety

All adverse events were transient and mild. The principal investigator found no association between adverse events and the intake of test foods. The incidence of adverse events did not differ between the groups (placebo: 63.0%; MCC1849: 73.7%; *p* > 0.1).

## 4. Discussion

We evaluated the effect of MCC1849 on subjective symptoms of physical condition in both sexes over a wide age range in this study. The results showed significantly fewer days with a stuffy nose and shorter durations of stuffy nose and sore throat in the MCC1849 group (Table 2). The alleviation of local symptoms was particularly evident. The MCC1849 group showed a trend toward a lower duration of feverishness, one of the systemic symptoms. Furthermore, MCC1849 significantly suppressed the onset of cold-like symptoms, including systemic and local symptoms. The effect on cold-like symptoms was consistent with a previous study of healthy young women who were susceptible to cold [12]. The same strain may have an effect only on local symptoms [14] or on both local and systemic symptoms, according to clinical trials [15]. Therefore, it is plausible that ingestion of MCC1849 has beneficial effects on both local and systemic symptoms.

We assessed each subjective symptom in addition to cold-like symptoms for detailed analyses. Furthermore, the duration and number of times of onset were calculated as well as the number of days and incidence. The efficacy of MCC1849 was consistent with a previous study [12], so we believe that our findings support the effect of MCC1849 on subjective symptoms of one’s physical condition. The immunomodulatory effect of heat-killed *L. paracasei*, to which MCC1849 belongs, has been reported in several studies. The administration of heat-killed *L. paracasei* KW3110 suppressed inflammatory CD4-positive T-cell expansion in vivo [16]. The effect of 200 billion heat-killed *L. paracasei* K71 was demonstrated on the cumulative days of local symptoms, including runny nose, stuffy nose, sneezing and sore throat in healthy adults [17]. Our study showed the effectiveness of fewer heat-killed *L. paracasei* than that study. Thus, MCC1849 could be added to various foods at low cost and in an easy-to-consume form. The induction of IL-12 was reported to differ between strains even if the strains were of the same species [18]. It has been speculated that different strains require different numbers of bacteria to have the same effect on physical condition.

The MCC1849 group had significantly fewer days of stuffy nose and shorter durations of stuffy nose and sore throat (Table 2). Stuffy nose and sore throat are familiar symptoms of the common cold, which develops due to inflammation as a defense response to bacteria or viruses [19]. MCC1849 can suppress these symptoms through its effects directly on the immune system. MCC1849 can induce the differentiation of IgA-positive B cells via follicular T cells and significantly increase the amount of antigen-specific IgA [10]. The role of IgA is to neutralize pathogen invasiveness or toxigenicity in mucosal surfaces [20]. A possible mechanism by which MCC1849 maintained physical condition was stimulating IgA production to block the invasion and infection by pathogens that cause the common cold. In addition, MCC1849 strongly induces IL-12 secretion [10]. IL-12 is mainly produced by dendritic cells and macrophages and promotes the differentiation of naive T cells into Th1 cells [21]. Th1 cells activate immune cells such as natural killer (NK) cells and macrophages [22]. NK cells and macrophages are involved in innate immunity. These cells prevent the invasion of pathogens or viruses by phagocytosis or induction of apoptosis [23]. Intake of MCC1849 might suppress subjective symptoms by activating NK cells and macrophages. The onset of cold-like symptoms, including systemic and local symptoms, was also decreased in the MCC1849 group (Table 2). The intake of heat-killed lactic acid bacteria was recently reported to suppress cold-like symptoms through the activation of plasmacytoid dendritic cells (pDCs) [15,24]. pDCs seem to be a key factor in immune responses by producing type I interferon and activating NK cells, B cells and killer T cells [25]. NK cells and killer T cells eliminate infected cells [23]. MCC1849 is also a postbiotic of *Lactobacillus*, so it could be speculated that MCC1849 activates pDCs. Thus, MCC1849 seems to be involved in both innate and acquired immunity. In short, ingestion of MCC1849 might contribute to suppression of the onset of the disease by IgA or innate immune cells, and even if the pathogens have entered the body, the duration of the symptoms is mildly suppressed by the activation of pDCs and macrophages.

We assessed body temperature as an objective measure of physical condition. The febrile state is defined as a temperature above 37.5 °C in Japan. No significant differences were found between groups in the effects of MCC1849 on body temperature above 37.5 °C (Table 3). We speculate that the effect of MCC1849 could not be fully evaluated due to the low incidence of the febrile state.

Since the incidence of the common cold increases in the winter, this study was conducted from winter to spring [1]. Adults are reported to catch colds two to four times a year, and the results of this study were consistent with this rate. Therefore, we consider this study appropriate for evaluating the subjective symptoms of physical condition. One problem was that there was a protocol deviation in randomization. Although this was not the prescribed method, the stratification factors were equally divided between the two groups. Thus, this error seemed not to affect the results of this study.

There were no side effects associated with the intake of test food for 24 weeks. A previous study similarly demonstrated the safety of MCC1849 [12]. MCC1849 has also obtained the self-affirmed “generally recognized as safe” (GRAS) notification for the intended use of conventional foods. MCC1849 is verified to be safe for a long time, so continuous intake of MCC1849 in daily foods is not expected to cause problems.

There were some limitations to this study. The participants were all healthy adults, so the effect of MCC1849 on children or sick individuals is unknown. Moreover, we did not measure immune parameters. Further studies evaluating the effects on subjective symptoms and immune parameters in the same study may elucidate the detailed mechanism of MCC1849.

## 5. Conclusions

Intake of 50 billion cells of MCC1849 by healthy adults of a wide age range suppressed subjective symptoms. Oral intake of MCC1849 may help maintain physical condition.

## Figures and Tables

**Table 1:** Baseline characteristics of the participants.

	Placebo	MCC1849	*p* Value
Age ^1,2^	44.48 ± 10.3	45.44 ± 9.6	0.514
Sex (male/female) ^3^	28/64	29/62	0.834
BMI ^1,2^	22.14 ± 3.4	21.95 ± 3.2	0.703
COVID-19 vaccination during the study period ^3^	63	59	0.601
Common cold in the past 3 years ^3^			0.927
None	4	4	
Once	36	39	
Twice	27	27	
3 times or more	25	21	

^1^ Values are mean ± standard deviation. ^2^ *p* values were calculated using the unpaired Student’s *t* test. ^3^ *p* values were calculated using the χ^2^ test.

**Table 2:** Days, times, duration and incidence of each symptom and cold-like symptoms.

Symptom	Total Number of Days of Each Symptom (Days) ^1^		*p* Value ^2^	Duration of Each Symptom (Days) ^1^		*p* Value ^2^	Total Number of Times of Each Symptom (Times) ^1^		*p* Value ^2^	Incidence of Each Symptom (*n*, %)		*p* Value ^3^
	Placebo	MCC1849		Placebo	MCC1849		Placebo	MCC1849		Placebo	MCC1849	
feverishness ^4^	0.86 ± 0.33	0.33 ± 0.13	0.105	0.38 ± 0.10	0.17 ± 0.06	0.094	0.44 ± 0.15	0.20 ± 0.07	0.125	18 (19.6)	10 (11.0)	0.107
chill ^4^	0.63 ± 0.24	0.74 ± 0.19	0.182	0.26 ± 0.08	0.43 ± 0.09	0.155	0.42 ± 0.16	0.40 ± 0.08	0.201	16 (17.4)	23 (25.3)	0.193
malaise ^4^	2.52 ± 0.85	1.46 ± 0.27	0.836	0.60 ± 0.12	0.58 ± 0.10	0.679	1.51 ± 0.47	0.96 ± 0.18	0.824	30 (32.6)	32 (35.2)	0.715
joint pain^4^	1.67 ± 0.78	1.51 ± 0.72	0.480	0.44 ± 0.12	0.49 ± 0.20	0.468	0.76 ± 0.35	0.35 ± 0.11	0.520	17 (18.5)	13 (14.3)	0.444
muscle pain ^4^	1.24 ± 0.70	0.54 ± 0.29	0.472	0.30 ± 0.10	0.34 ± 0.16	0.491	0.58 ± 0.33	0.14 ± 0.05	0.472	11 (12.0)	8 (8.8)	0.483
sneeze/runny nose ^4^	5.21 ± 1.73	3.19 ± 0.88	0.493	1.17 ± 0.20	0.91 ± 0.15	0.534	1.52 ± 0.33	1.32 ± 0.32	0.467	40 (43.5)	36 (39.6)	0.591
stuffy nose ^4^	4.19 ± 1.05	1.49 ± 0.36	0.043	1.23 ± 0.22	0.64 ± 0.14	0.038	1.47 ± 0.35	0.64 ± 0.15	0.072	36 (39.1)	24 (26.4)	0.066
sore throat ^4^	2.09 ± 0.61	1.13 ± 0.30	0.064	1.02 ± 0.21	0.68 ± 0.20	0.046	0.79 ± 0.21	0.44 ± 0.11	0.084	33 (35.9)	21 (23.1)	0.058
Cough ^4^	1.48 ± 0.67	1.23 ± 0.48	0.617	0.71 ± 0.21	0.52 ± 0.17	0.556	0.46 ± 0.25	0.41 ± 0.16	0.677	16 (17.4)	13 (14.3)	0.565
cold-like symptoms ^5^	23.19 ± 4.83	12.84 ± 3.38	0.037	6.65 ± 1.77	4.20 ± 1.92	0.019	3.20 ± 0.46	2.28 ± 0.34	0.110	64 (69.6)	52 (57.1)	0.081

^1^ Values are mean ± standard error. ^2^ *p* values were calculated using Wilcoxon rank sum test. ^3^ *p* values were calculated using the χ^2^ test. ^4^ The onset was defined as a symptom score of three or higher. ^5^ The onset was defined as a total score of all symptoms of six or higher.

**Table 3:** Days and incidence of body temperature above 37.5 °C.

	Total Number of Days (days) ^1^		*p* Value ^2^	Incidence (*n*, %)		*p* Value ^3^
	Placebo	MCC1849		Placebo	MCC1849	
body temperature above 37.5 °C	0.16 ± 0.067	0.14 ± 0.049	0.981	9 (9.78)	9 (9.89)	0.981

^1^ Values are mean ± standard error. ^2^ *p* values were calculated using the Wilcoxon rank sum test. ^3^ *p* values were calculated using the χ^2^ test.

## Data Availability

The data presented in this study can be found in this published article.
